# Rapid response systems

**DOI:** 10.4103/0972-5229.42561

**Published:** 2008

**Authors:** Ken Hillman

**Affiliations:** **From:** University of New South Wales; Critical Care Services, Sydney South West Area Health Service, Sydney, Australia

**Keywords:** Cardiac arrest, medical emergency team, outreach, patient safety, rapid response teams

## Abstract

Intensive care medicine was for many years practiced within the four walls of an intensive care unit (ICU). Evidence then emerged that many serious adverse events in hospitals were preceded by many hours of slow deterioration, resulting in multi-organ failure and potentially preventable admissions to the ICU. Ironically, these admissions may have been prevented if the skills within the ICU had been available to the patient on the general ward at an earlier stage. The concept of a Medical Emergency Team (MET) was developed to enable staff from the ICU to rapidly identify and respond to serious illness at an earlier stage and, hopefully, prevent serious complications. Since then, other forms of rapid response and outreach systems have been developed. Increasingly, physicians working in ICUs can see the benefit of the early management of serious illness in order to improve patient outcome.

Intensive care medicine arguably began in Copenhagen in 1952, when victims of poliomyelitis were artificially ventilated in order to sustain life until the disease abated.[1] As a result, the mortality was reduced from 89% to 40% - a remarkable achievement.

Soon the skills learnt in managing these patients was applied to other seriously ill patients, including patients with severe trauma, serious infections and other diseases such as tetanus. Patients were now able to be kept alive while their underlying disease was either actively treated or abated in the course of time. The development of intensive care also meant that complex surgery was able to be performed. Specialties such as cardiac surgery, vascular surgery and neurosurgery were able to be developed as a result of the parallel development of intensive care medicine.

The actual space that defines an intensive care unit (ICU) was essential to the development of the specialty of intensive care medicine. Specific training programs were developed in the specialty, firstly for nursing staff and then for physicians. The walls of the ICU nurtured the specialty. Monitoring of the seriously ill with specific machines was developed. Artificial ventilation, dialysis and inotropes were used to support vital functions. The specialty would not have developed if these devices and interventions had to be transferred to the general wards. However, the security and sense of accomplishment may have, at the same time, contained our thinking to within the four walls of the ICU. For many years patients were considered either sick enough to benefit from being in ICU or well enough to be able to be treated on the general wards. It was black or white. And yet, at the same time, our research clearly demonstrated that serious illness often began long before admission to the ICU. In fact, the specialty of intensive care often simply involved treating multi-organ failure as a result of untreated ischemia and hypoxia.

While the management of the seriously ill within the four walls of the ICU improved markedly, the standard of care for at-risk patients outside the ICU was questionable. Over 80% of in-hospital cardiac arrests are preceded by serious abnormalities in vital signs within eight hours of the arrest.[2,3]

Up to 40% of ICU admissions are potentially avoidable[4] and approximately half of those patients had received substandard care before admission to the ICU. Serious adverse events, including deaths, occur in up to 17% of hospital patients and approximately 70% of those are preventable.[5] Almost half of all patients who die without a "not for resuscitation" (NFR) order have serious and potentially reversible abnormalities in their vital signs in the 24 hours before death.[6]

Interestingly, early studies in patients who were admitted to the ICU hinted at the effects of delayed resuscitation. It was noted that the APACHE score was influenced by pre-ICU care – a phenomenon called “lead-time” bias.[7] The concept of the "golden hour" emphasizes one of the most important aims in the management of the critically ill – to rapidly restore oxygenated blood flow to tissues. There is good evidence that the beginnings of multi-organ dysfunction syndrome (MODS)[8–12] long before admission to the ICU.

Despite this knowledge, much of the research conducted by intensive care specialists is around managing the seriously ill after they have been admitted to the ICU, such as defining ideal tidal volumes and selecting the best inotropes or antibiotics; and searching for magic bullets after MODS has been established.[13]

Paradoxically, there has been little research evaluating systems for early care of the seriously ill, before irreversible organ failure has occurred. For example, it was fashionable at one time to conduct research into the effect of supranormal oxygen delivery after the patient was admitted to the ICU. Careful reading of these studies suggest that this approach amounted to “too much, too late”.[14–18] It could be concluded from these articles that early restoration of the intravascular volume may have been more effective than late supranormal oxygen delivery. For example, when goal directed therapy was initiated at an earlier stage in the emergency department, patient outcome improved.[19]

In order to improve patient outcome it seems logical to recognize seriously ill patients early and to rapidly resuscitate them. This may seem logical but it involves establishing a hospital-wide system. Something health has not necessarily had a lot of experience with. The only hospital-wide system in many organizations is the cardiac arrest team which has not improved mortality in the almost 50 years since the concept was first implemented.[20] Hospitals, and indeed medical training, are built around the long tradition of individual physicians being responsible for the care of individual patients.

To identify and manage patients within a different paradigm that crosses all the usual hospital silos is difficult.

Even identifying at-risk patients is difficult[4] as is responding to their needs with staff skilled in all aspects of resuscitation is a challenge.[4,21]

Nurses have traditionally recorded deteriorating signs and noted patients who were “going off” but have not been empowered, nor trained to act on those signs. They often rely on junior doctors who, themselves, have had little undergraduate training in advanced resuscitation.[22,23] The specialist responsible for the patient's care is not always immediately available nor trained in advanced resuscitation.

Another reason for poor management of at-risk patients is related to the hierarchical medical system where problems in acute hospitals are passed up through levels of seniority. While individual specialists formally consult others when necessary, this process often takes hours or even days and potentially seriously ill patients require immediate attention.

Trauma systems were the first to attempt to construct care around patient needs from the first point of immediate care at the site of injury, to transport to hospital, resuscitation in the emergency department, management in hospital and rehabilitation.[24–27] Ironically, often immediate and appropriate care is delivered better for the seriously ill in the community than it is in acute hospitals.

A MET was first established in 1989 at Liverpool Hospital in Sydney, Australia, in an attempt to recognize seriously ill patients early and to respond rapidly to their needs.[28] The cardiac arrest team was renamed the MET and a set of criteria based on abnormal vital signs and observations were developed as triggers [Table 1].[29]

**Table 1:** Criteria for calling the medical emergency team

Acute changes in	Physiology
Airway	Threatened
Breathing	All respiratory arrests
	Respiratory Rate <5
	Respiratory Rate >36
Circulation	All cardiac arrests
	Pulse rate <40
	Pulse rate >140
	Systolic blood pressure <90 mmHg
Neurology	Sudden fall in level of consciousness (Fall in GCS of >2 points)
	Repeated or prolonged seizures
Other	Any patient who you are seriously worried about that does not fit the above criteria

The MET concept is based on recognizing seriously ill and at-risk patients early with the aim of preventing death and serious adverse events. The concept is a system with at least three separate components - criteria defining an at-risk patient; a rapid response by staff with appropriate skills, knowledge and experience; and ways of monitoring the system and closing the loop with that information so that continuous quality improvement occurs.

For monitoring to occur, data must be collected.[30] Some data that can be used for monitoring include deaths, cardiac arrests and unanticipated admissions to the ICU. In order to exclude patients who are terminally ill, patients who have an explicit NFR entry are excluded and the remainder are called “unexpected.” “Unexpected” admissions to the ICU are those who are mainly from the general wards and do not include patients from emergency departments or operating suites. However, they may include patients from areas such as diagnostic suites or coronary care units.

In order to facilitate the organization using the data for quality assurance purposes, clinical notes can be scanned to see if any MET criteria were present in the 24 hours before the event. The data should then inform all levels of the organization as a quality assurance tool.

Other ways of monitoring the system include presenting details of MET activity at regular intervals. This would include not only the number of calls, but the site of the call, the nature of the intervention, how long each call took and the patient outcome.

The concept of early identification of at-risk patients, together with a rapid response has now been adapted in many ways. The criteria may vary slightly and the response may be multi-tiered, with perhaps the home team or attending nurse being the first response and then, if the patient requires a higher level of support, a more experienced team is called. Examples of these variations include the patient at-risk team (PART)[31] and the modified early warning score (MEWS).[32]

Then there is the concept of outreach,[33] which usually involves staff who have been trained in caring for the seriously ill, playing a proactive role in the general wards, which may decrease the need for emergency calls using education across the hospital and playing a consultative role in the care of the seriously ill.

There have been several studies[34–36] evaluating the impact of early response systems. In three important before and after studies, the introduction of the MET has been associated with a reduction in cardiac arrests and death rates as well as a reduction in intensive care and hospital stay.

A case controlled study[37] demonstrated reduced mortality as well as the incidence of unanticipated admission rates to ICUs. The system has also improved postoperative care.[38,39]

The outreach system has also resulted in improved patient care across a large number of clinical indicators.[38,40–44]

A large cluster randomized trial involving 23 Australian hospitals failed to demonstrate a difference between the MET and control hospitals (MERlT study).[45] However, it did provide insight into the challenges of effective implementation of a system across an entire hospital. Less than half of all patients with the MET criteria actually had a call made. Approximately the same number had no vital signs recorded before serious adverse events occurred. Moreover, there was such a variation of outcomes in the MET hospitals that statistical significance would have only been possible if more than 100 hospitals had been recruited.

No one would propose that we do not treat serious illness as early as possible. However, the challenge for hospitals is to effectively implement a system across the entire organization and this is something health has traditionally had little experience.

Because early warning systems make sense they have now been implemented in many hospitals in Europe, North America and Australasia. They will almost certainly become, in one way or another, a critical part of all acute hospitals.
